# Pancreatic β-Cell Death due to Pdx-1 Deficiency Requires Multi-BH Domain Protein Bax but Not Bak*

**DOI:** 10.1074/jbc.M115.705293

**Published:** 2016-05-02

**Authors:** Juan Sun, Li-qun Mao, Kenneth S. Polonsky, De-cheng Ren

**Affiliations:** From the ‡ Department of Medicine, University of Chicago, Chicago, Illinois 60637 and; § Department of Microbiology & Immunology, Rosalind Franklin University of Medicine and Science, North Chicago, Illinois 60064

**Keywords:** apoptosis, Bax, beta cell (β-cell), cell death, diabetes

## Abstract

Diabetes develops in *Pdx1*-haploinsufficient mice due to an increase in β-cell death leading to reduced β-cell mass and decreased insulin secretion. Knockdown of *Pdx1* gene expression in mouse MIN6 insulinoma cells induced apoptotic cell death with an increase in Bax activation and knockdown of Bax reduced apoptotic β-cell death. In *Pdx1* haploinsufficient mice, *Bax* ablation in β-cells increased β-cell mass, decreased the number of TUNEL positive cells and improved glucose tolerance after glucose challenge. These changes were not observed with Bak ablation in *Pdx1-*haploinsufficient mice. These results suggest that Bax mediates β-cell apoptosis in *Pdx1*-deficient diabetes.

## Introduction

Pancreas and duodenal homeobox-1 (Pdx1)^3^ plays an important role in pancreas development and in maintaining β-cell function and survival. Previous studies from our laboratory and others have shown that heterozygous *Pdx1*^+/−^ mice develop diabetes due to decreased β-cell mass. Islets from *Pdx1*^+/−^ mice are more susceptible to apoptosis (1–3).

In mammalian cells, apoptosis is mainly regulated by two signaling pathways: the extrinsic or death receptor pathway and the intrinsic or mitochondrial pathway. The extrinsic signaling pathway that initiates apoptosis involves transmembrane receptor-mediated interactions including FAS ligand-FAS/APO1, TNF-TNF receptors, and TRAIL-TRAIL receptors. In the intrinsic pathway, the BCL-2 family BH3-only molecules, Bid, Bim, and Puma, convey apoptotic signals to trigger homo-oligomerization of multidomain proapoptotic Bax and Bak, which in turn permeabilize mitochondria, leading to the efflux of cytochrome *c*, the assembly of the apoptosome, and the activation of caspases which mediate apoptosis (4). During apoptosis, Bax translocates from the cytosol to insert into the outer membrane of mitochondria, and both Bax and Bak can convert from the non-activated to the activated conformation (5).

We have shown that both Bim and Puma mediate Pdx1 deficiency induced β-cell death (6). The present study was undertaken to determine whether the multi-BH domain protein Bax and Bak, downstream molecules of Bim and Puma, also mediate pancreatic β-cell death associated with Pdx1 suppression.

## Experimental Procedures

### 

#### 

##### MIN6 Cell Culture, Quantification of mRNA Levels, and Lentivirus-mediated shRNA Expression

MIN6 cell culture, RNA isolation, and first-strand cDNA synthesis, and preparation of pLKO.1-Pdx1 shRNA lentivirus were performed as previously described (6). TaqMan assay numbers were: Hmbs, Mm00660262; Pdx1, Mm00435565; Bax, Mm00432051; and Bak, Mm00432045. The pLKO-Bax shRNA (RMM4533), Bak shRNA (RMM4534) were purchased from Thermo Scientific. Lentivirus was added to the medium on day 1. The blots were probed with antibodies against Pdx1 (07-696; Millipore), cytochrome *c* (mouse6H2.B4, Millipore), Actin (A-2066; Sigma), Bax (6A7) (2281-MC-100; Trevigen), Bax (N20) (SC493, Santa Cruz Biotechnology) and Bak (06536, Millipore). To detect BAX activation, immunoprecipitation (IP) was performed using 1% CHAPS buffer (1% CHAPS, 142.5 mm KCl, 2 mm CaCl_2_, 20 mm Tris-Cl, pH 7.4). Anti-6A7 IP was performed using 1% CHAPS buffer. Antibody detection was accomplished using enhanced chemiluminescence (PerkinElmer) and LAS-3000 Imaging system (FUJIFILM).

##### Quantitation of Cell Death

Cell death was quantified by propidium iodide (PI) staining (7) followed by flow cytometric analyses using a FACS Caliber (BD Bioscience) and FlowJo software.

##### Flow Cytometric Analysis of Mitochondrial Membrane Potential

Mitochondrial membrane potential was assessed by TMRE (tetramethylrhodamine, ethyl ester) staining followed by flow cytometric analysis (8). TMRE enters cells and reversibly accumulates in the highly negatively charged mitochondrial matrix according to the Nernst equation, allowing the potential to be measured.

##### Immunofluorescence Analysis of Cytochrome c

After 4 days of treatment with lentiviral control or Pdx1 shRNA, MIN6 cells were fixed for 15 min in 4% paraformaldehyde, permeabilized with 0.5% Triton X-100/PBS for 5 min and then incubated for 1 h in a 5% BSA/PBS blocking solution. Then cells were incubated overnight at 4 °C with a mouse monoclonal anti-cytochrome *c* IgG (Pharmingen) followed by exposure to a goat anti-mouse Alexa488-conjugated secondary antibody (Invitrogen). Images were obtained on an Evos microscope (Advanced Microscopy Group).

##### Tamoxifen Administration

In this study, over a 5-day period, 4-week-old male mice were injected intraperitoneally with 3 doses of 2.5 mg of tamoxifen (Sigma, T5648) freshly dissolved in corn oil at 10 mg/ml (9).

##### In vivo Characterization of Mice

The *Pdx1*^+/−^ mice have been previously described (4). *Bax*^F/F^*Bak*^−/−^ mice (10) were provided by Dr. Emily Cheng (Memorial Sloan-Kettering Cancer Center) and *MIP-Cre/ERT* mice (9) by Louis Philipson (University of Chicago). Male mice were fed a high-fat diet containing 42% fat (Harlan Laboratories Inc.) from 5 weeks of age and provided with water *ad libitum* as previously described (11). The relative β-cell area was measured from anti-insulin-stained pancreas sections counterstained with hematoxylin using ImageJ software. TUNEL and Ki-67 staining were performed as previously described (11). More than 20000 β-cells and 300 islets were counted after TUNEL and Ki-67 staining and at least three mice were counted per group. All animal experiments in this study were performed under protocols approved by the University of Chicago Animal Studies Committee.

##### Imaging Studies of Pancreatic Islets

Formalin-fixed pancreas sections underwent antigen retrieval in boiling citrate buffer (pH 6.0) for 10 min before labeling with antibodies against insulin (A0564; DAKO), glucagon (G2654; Sigma-Aldrich), and DAPI (P-36931; Invitrogen).

##### Statistical Analysis

The 2-tailed unpaired Student's *t* test was used to assess the statistical significance of differences between 2 sets of data. Differences were considered significant when *p* < 0.05. In all experiments, the number of asterisks is used to designate the following levels of statistical significance: ***, *p* < 0.001; **, *p* < 0.01; *, *p* < 0.05 compared with control group or wild type (WT) group. ####, *p* < 0.001; ##, *p* < 0.01; #, *p* < 0.05 compared with Pdx1 KD or *Pdx1*^+/−^ group. Results are presented as mean ± S.E.

## Results

### 

#### 

##### Pdx1 Suppression Activates Bax in MIN6 Cells

**FIGURE 1.**
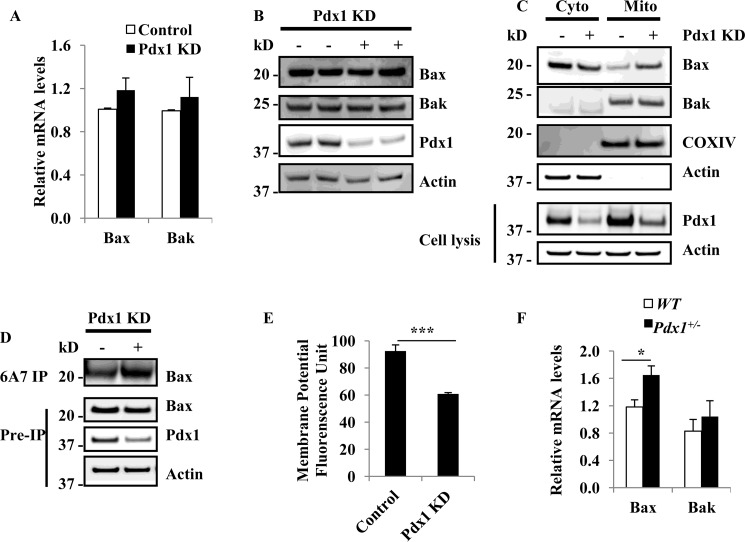
**Pdx1 knockdown induces Bax activation in MIN6 cells and islets.** *A*, Bax and Bak mRNA levels in control and Pdx1 KD cells. 3 days after Pdx1 KD in MIN6 cells, Bax and Bak mRNA levels were not different between control and Pdx1 KD cells (*n* = 3). *B*, Western blot of Pdx1 KD cells. 3 days after Pdx1 KD in MIN6 cells, immunoblot analysis was performed to determine Pdx1, Bax, and Bak in Pdx1 KD MIN6 cells. *C*, Bax nuclear/cytosolic translocation in Pdx1 KD MIN6 cells. 3 days after Pdx1 shRNA lentivirus infection, cytosolic and nuclear proteins were analyzed by Western blot. *D*, immunoprecipitate of Bax. 3 days after Pdx1 KD in MIN6 cells, cells were lysed in 1% CHAPS and then immunoprecipitated with the 6A7 anti-BAX antibody. Immunoprecipitates were analyzed by anti-BAX (N20) immunoblots. *E*, effects of Pdx1 on ΔΨ_m_. MIn6 cells were treated with Pdx1 shRNA lentivirus for 0, 4 days. Cells were stained with TMRE dye to measure ΔΨ_m_. ***, *p* < 0.001. *F*, Bax and Bak mRNA levels in islets. Bax and Bak mRNA levels were measured by real time reverse transcription-PCR in islets from 5–6-week-old male Pdx1^+/−^ mice on normal chow (*n* = 3–6). *, *p* < 0.05 compared with wild type (WT) mice.

Pdx1 KD MIN6 cells did not demonstrate a significant increase in *Bax* and *Bak* mRNA (Fig. 1*A*) or protein levels when compared with control cells (Fig. 1*B*). Since Bax is located in the cytosol until activated by a diversity of stress stimuli to induce cell death through translocation to mitochondria, cellular cytosol, and mitochondrial fractions were extracted to determine if Bax subcellular fractions were altered by Pdx1 suppression. Pdx1 KD induced an accumulation of Bax protein in the mitochondrial fraction (Fig. 1*C*) but no change in Bak protein levels. Protein levels of cytochrome *c* oxidase subunit IV (COX IV), a mitochondrial marker, were similar in Pdx1 KD and control cells (Fig. 1*C*).

Bax conformation was also examined using the monoclonal antibody 6A7, which only recognizes the N-terminal epitope of Bax (12). The results showed that the amount of Bax precipitated by 6A7 anti-Bax antibody was increased in Pdx1 KD cells (Fig. 1*D*). To determine the effect of Bax mitochondrial translocation, mitochondrial membrane potential (ΔΨ_m_) was measured by quantifying the average mitochondrial fluorescence intensity of TMRE. TMRE uptake into mitochondria was decreased from 92.4 ± 4.5% in control cells to 60.7 ± 1.0% in Pdx1 KD cells (*p* < 0.001) indicating Pdx1 KD significantly decreased mitochondrial membrane potential (Fig. 1*E*). Bax and Bak mRNA levels were also examined in pancreatic islets isolated from 5–6 weeks old *Pdx1*^+/−^ mice. mRNA levels of Bax were increased in islets from *Pdx1*^+/−^ mice (*p* < 0.05) (Fig. 1*F*). Bak mRNA levels did not increase.

##### Bax Suppression Reduced β-Cell Apoptosis Induced by Pdx1 KD in MIN6 Cells

**FIGURE 2.**
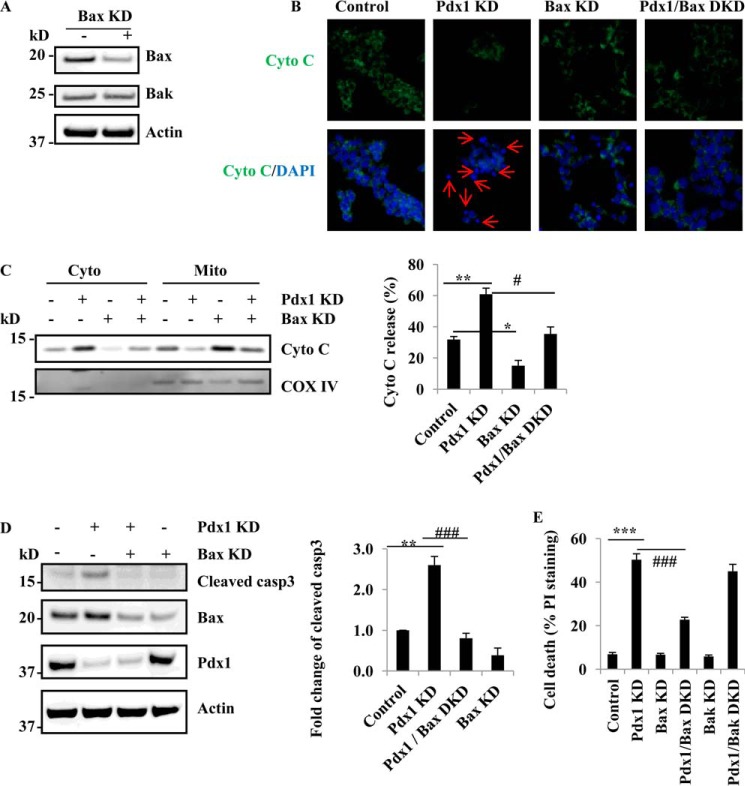
**Bax is necessary for β-cell apoptosis induced by Pdx1 suppression.** *A*, Bax protein levels in Bax KD cells. 3 days after Bax knockdown, immunoblot of Bax, Bak, and actin in Bax KD MIN6 cells. *B*, Bax KD inhibits the translocation of cytochrome *c* in Pdx1/Bax DKD MIN6 cells. Fluorescence microscopy of MIN6 cells 4 days after exposure to Pdx1 and Bax lentivirus. *Green* represents cytochrome *c* immunostaining, and *blue* is DAPI staining. *Red arrows* indicate apoptotic cells that have lost cytochrome *c*. Scale bar, 20 μm. *C*, Bax inhibits cytochrome *c* release induced by Pdx1 KD. 3 days after Bax and Pdx1 KD in MIN6 cells, the levels of cytochrome *c* in the cytosol and mitochondria fractions are determined by immunoblot. Bar graphs represent quantification using densitometry of the relative amounts of the indicated proteins determined by Western blots (*n* = 3). *, *p* < 0.05; **, *p* < 0.01; and #, *p* < 0.05. *D*, immunoblot of Pdx1, Bax and cleaved caspase3 in Pdx1/Bax DKD MIN6 cells. Bar graphs represent quantification using densitometry of the relative amounts of the indicated proteins determined by Western blots. **, *p* < 0.01 and ###, *p* < 0.001. *E*, measurement of cell death. 4 days after Pdx1/Bax and Pdx1/Bak DKD in MIN6 cells, cell death was determined by PI-staining (*n* = 3). ***, *p* < 0.01 and ###, *p* < 0.001. Values are mean ± S.E.

To define the functional effects of changes in Bax expression on pancreatic β-cell death after Pdx1 suppression, shRNA was used to knock down Bax in MIN6 cells. Lentiviral Bax shRNA suppressed Bax expression by more than 60% and did not affect Bak expression (Fig. 2*A*).

In Pdx1 KD MIN6 cells, Bax suppression inhibited the increase in cytochrome *c* release from mitochondria, the key event in activating apoptosis (Fig. 2*B*). Western blot also showed that Pdx1 induced an increase of cytochrome *c* release from mitochondria into the cytosol (Fig. 2*C*). Bax KD inhibited the cytochrome *c* release induced by Pdx1 KD (Fig. 2*C*). Pdx1 KD increased cytochrome *c* by 93% compared with control group (*p* < 0.01) (Fig. 2*C*). However, Bax KD significantly inhibited cytochrome *c* release by 42% in Pdx1 KD cells (Fig. 2*C*). Pdx1 KD increased cleaved caspase 3 protein by 150% compared with control cells (*p* < 0.001, Fig. 2*D*). In Bax/Pdx1 double knockdown (DKD) cells the cleaved caspase 3 protein levels were significantly lower than in Pdx1 KD cells (80% *versus* 250%, *p* < 0.001) (Fig. 2*D*). Furthermore, following Pdx1 KD, 50.2 ± 2.7% of the MIN6 cells took up the PI stain. In the Pdx1/Bax DKD group, only 22.7 ± 1.1% (*p* < 0.001 compared with Pdx1 alone) took up the PI stain indicative of a 44% increase in cell viability (Fig. 2*E*). Bak knockdown had no effect on β-cell death induced by Pdx1 suppression (Fig. 2*E*).

##### Effect of Bax Ablation in Adult Pdx1^+/−^ Mice

**FIGURE 3.**
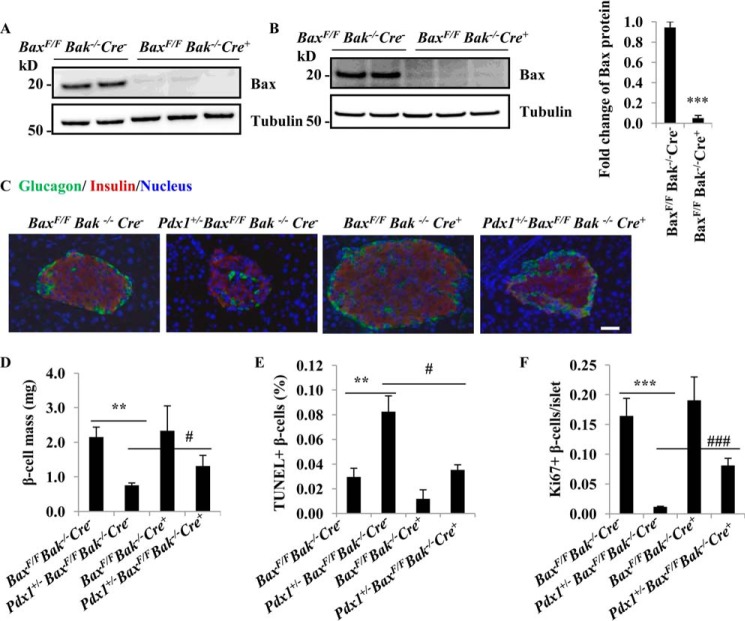
**Bax ablation protects β-cells in adult *Pdx1*^+/−^*Bax*^F/F^*Bak*^−/−^*Cre*^−^ mice.** *A* and *B*, Western blot of Bax in islets from 4-month old mice with normal chow (*A*) and high fat diet (*B*). *C*, islet morphology in adult mouse after 12 weeks on a high fat diet. Anti-insulin and anti-glucagon antibodies were used to stain β-cells (*red*) and α cells (*green*) respectively. Scale bar, 20 μm. *D*, histological analysis of pancreatic islets and quantitation of group data for β-cell mass (*n* = 3–5 per group). **, *p* < 0.01 compared with the *Bax*^F/F^*Bak*^−/−^*Cre*^−^ mice. #, *p* < 0.05 compared with *Pdx1*^+/−^*Bax*^F/F^*Bak*^−/−^*Cre*^−^ mice. *E*, TUNEL labeling of adult pancreatic β-cells. Quantitative TUNEL data are shown. **, *p* < 0.01 compared with the *Bax*^F/F^*Bak*^−/−^*Cre*^−^ mice. #, *p* < 0.05 compared with *Pdx1*^+/−^*Bax*^F/F^*Bak*^−/−^*Cre*^−^ mice. Original magnification, ×200. *F*, Ki-67 staining of β-cells. ***, *p* < 0.001 compared with the *Bax*^F/F^*Bak*^−/−^*Cre*^−^ mice. ###, *p* < 0.001 compared with *Pdx1*^+/−^*Bax*^F/F^*Bak*^−/−^*Cre*^−^ mice. All group data are mean ± S.E. of *n* = 3.

To determine the effects of *Bax* deficiency on β-cell death *in vivo*, we used mice in which *Bax* is conditionally deleted in islets using *MIP-Cre/ERT* (here refers to Cre) on a *Bak*^−/−^ background. The expression of Bax protein in islets from mice fed either a normal chow or a high fat diet was decreased to the extent that it became almost undetectable after tamoxifen treatment (Fig. 3, *A* and *B*). Bax protein levels in islets from *Bax*^F/F^*Bak*^−/−^*Cre*^+^ mice after 4 months on a high fat diet were 5% of the levels in islets from *Bax*^F/F^*Bak*^−/−^*Cre*^−^ mice. β-cell mass was reduced by 65% (*p* < 0.01) in *Pdx1*^+/−^*Bax*^F/F^*Bak*^−/−^*Cre*^−^ mice and the islets contained reduced numbers of β-cells (Fig. 3*C*). These islets also demonstrated abnormal architecture in that α cells were distributed throughout the islets compared with islets from *Bax*^F/F^*Bak*^−/−^*Cre*^−^ mice that had a central core of β-cells ringed by a mantle of α cells (Fig. 3*C*). The *Pdx1*^+/−^*Bax*^F/F^*Bak*^−/−^*Cre*^+^ mice showed an increase in β-cell mass compared with *Pdx1*^+/−^*Bax*^F/F^*Bak*^−/−^*Cre*^−^ mice by 73% (*p* < 0.01) (Fig. 3*D*). The proportion of β-cells that demonstrated TUNEL labeling decreased significantly from 0.08 ± 0.01% in *Pdx1*^+/−^*Bax*^F/F^*Bak*^−/−^*Cre*^−^ mice to 0.035 ± 0.004% in *Pdx1*^+/−^*Bax*^F/F^*Bak*^−/−^*Cre*^+^ mice (*p* < 0.05) (Fig. 3*E*). To determine whether there was also an effect of *Bax* ablation on β-cell proliferation, the islets were stained for the proliferative marker Ki-67. Proliferation of β-cells was decreased in *Pdx1*^+/−^*Bax*^F/F^*Bak*^−/−^*Cre*^−^ islets and was significantly increased following *Bax* ablation in *Pdx1*^+/−^*Bax*^F/F^*Bak*^−/−^*Cre*^+^ islets (*p* < 0.001) (Fig. 3*F*).

##### Pdx1^+/−^ Mice with Bax Gene Ablation in Islets Have Improved Glucose Tolerance

**FIGURE 4.**
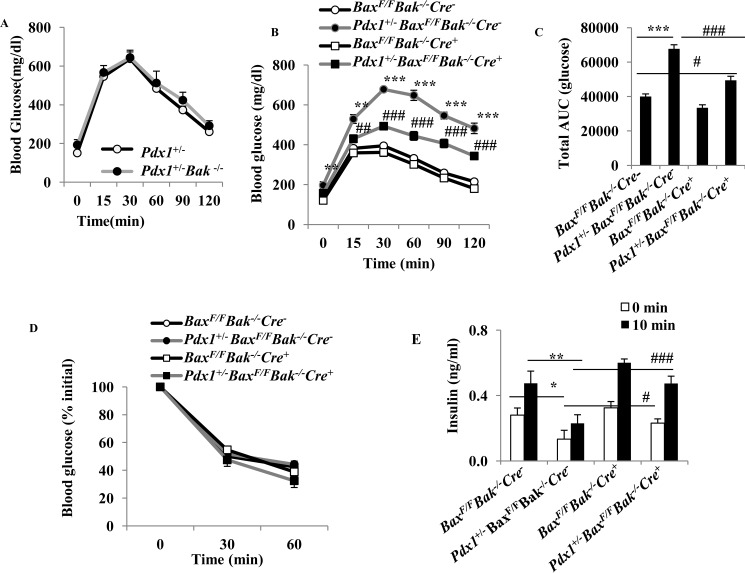
**Bax gene ablation in islets inhibits diabetes in *Pdx1*^+/−^*Bax*^F/F^*Bak*^−/−^*Cre*^−^ mice.** *A* and *B*, blood glucose levels after intraperitoneal injection of dextrose (1 g/kg) in the male mice on a HFD for 13 weeks. **, *p* < 0.01; ***, *p* < 0.001 compared with the *Bax*^F/F^*Bak*^−/−^*Cre*^−^ mice. ##, *p* < 0.01; ###, *p* < 0.001 compared with *Pdx1*^+/−^*Bax*^F/F^*Bak*^−/−^*Cre*^−^ mice (*n* = 8–12). *C*, area under the blood glucose curves (AUC) using the data from A in the 4 mouse groups designated. ***, *p* < 0.001 compared with the *Bax*^F/F^*Bak*^−/−^*Cre*^−^ mice. #, *p* < 0.05 compared with *Bax*^F/F^*Bak*^−/−^*Cre*^−^ mice, ###, *p* < 0.001 compared with *Pdx1*^+/−^*Bax*^F/F^*Bak*^−/−^*Cre*^−^ mice. *D*, glucose levels in response to 0.75 units/kg body weight insulin in the 4 male mouse groups designated on a HFD for 14 weeks (*n* = 8–10). *E*, insulin levels measured fasting and 10 min after intraperitoneal dextrose in mice on a HFD for 13 weeks. *, *p* < 0.05; **, *p* < 0.01 compared with the *Bax*^F/F^*Bak*^−/−^*Cre*^−^ mice. #, *p* < 0.05; ###, *p* < 0.001 compared with *Pdx1*^+/−^*Bax*^F/F^*Bak*^−/−^*Cre*^−^ mice. Values are mean ± S.E.

Breeding Pdx1^+/−^ mice on a *Bak*^−/−^ background did not result in an improvement in glucose tolerance (Fig. 4*A*). *Pdx1*^+/−^*Bax*^F/F^*Bak*^−/−^*Cre*^−^ mice fed high-fat diet developed increased fasting blood glucose and impaired glucose tolerance (Fig. 4*B*). However, *Pdx1*^+/−^*Bax*^F/F^*Bak*^−/−^*Cre*^+^ mice exhibited significantly lower fasting blood glucose and improved glucose tolerance compared with *Pdx1*^+/−^*Bax*^F/F^*Bak*^−/−^*Cre*^−^ (Fig. 4*B*). *Pdx1*^+/−^*Bax*^F/F^*Bak*^−/−^*Cre*^+^ mice showed significantly improved but not normal glucose tolerance compared with *Bax*^F/F^*Bak*^−/−^*Cre*^−^ mice (Fig. 4*B*). The area under the blood glucose curve (AUC) decreased 27% in *Pdx1*^+/−^*Bax*^F/F^*Bak*^−/−^*Cre*^+^ mice compared with *Pdx1*^+/−^*Bax*^F/F^*Bak*^−/−^*Cre*^−^ mice (*p* < 0.001). The AUC in *Pdx1*^+/−^*Bax*^F/F^*Bak*^−/−^*Cre*^+^ mice is higher compared with that in *Bax*^F/F^*Bak*^−/−^*Cre*^−^ mice (*p* < 0.05) (Fig. 4*C*). The reduction in blood glucose after insulin administration was similar in the four groups of mice (Fig. 4*D*) indicating that there were no differences in insulin sensitivity. Insulin levels were decreased in the *Pdx1*^+/−^*Bax*^F/F^*Bak*^−/−^*Cre*^−^ mice under basal conditions and following glucose challenge compared with *Bax*^F/F^*Bak*^−/−^*Cre*^−^ mice (Fig. 4*E*). In comparison, insulin concentrations were increased in *Pdx1*^+/−^*Bax*^F/F^*Bak*^−/−^*Cre*^+^ mice (Fig. 4*E*).

## Discussion

We have previously demonstrated that reduced β-cell mass is an essential component of the diabetic phenotype in the *Pdx1*-deficient mouse (2), and Bim and Puma mediate β-cell death induced by *Pdx1*-deficiency (6). In the intrinsic apoptosis pathway, both Bim and Puma can induce Bax or Bak activation and cause cell apoptosis (12). In the present studies, we demonstrated that pdx1 KD induces N-terminal conformational change in Bax and translocation of Bax to the mitochondria leading to its activation, alterations in mitochondrial membrane potential and cytochrome *c* release. Knockdown of Bax significantly reduced β-cell apoptosis and increased β-cell survival in Pdx1 deficient cells. In contrast, deficiency of Bak had no impact on these processes. Results obtained in the *Pdx1*^+/−^ mouse were consistent with the *in vitro* results. Bak^−/−^ alone had no effect on glucose tolerance in the *Pdx1*^+/−^ mouse. The reduction in the expression of Bax in islets preserved β-cell mass as a result of a reduction in β-cells apoptosis and an increase in proliferation of β-cells in *Pdx1*^+/−^*Bax*^F/F^*Bak*^−/−^*Cre*^+^ mice fed a high-fat diet compared with *Pdx1*^+/−^*Bax*^F/F^*Bak*^−/−^*Cre*^−^ mice. These data suggest that Bax rather than Bak is the molecule downstream of Bim and Puma that plays a critical role in mediating β-cell apoptosis induced by *Pdx1* deficiency. These results are consistent with other studies. One study showed that Bim, Puma, and Bax are required for β-cell apoptosis triggered by high glucose. Loss of the BH3-only proteins Bim or Puma, or loss of Bax markedly protected islets from glucose toxicity (13). Furthermore, in human type 2 diabetic subjects, expression levels of Bim, Puma, and Bax are increased when compared with non-diabetic donors (14). These results indicate that Bcl-2 family members involved in regulating the apoptotic pathway are implicated in β-cell death induced by Pdx1 deficiency, and also suggest possible targets to reduce β-cell apoptosis in diabetic syndromes associated with reduced Pdx1 such as MODY4.

Our approach is based on the following line of reasoning. Bak deficiency in the context of Cre expression in *Bax*^F/F^*Bak*^+/+^*Cre*^+^ mice should have no impact on these beta cell parameters. We base this conclusion on the observation that the response of *Pdx1*^+/−^*Bak*^−/−^ mice to glucose challenge is the same as *Pdx1*^+/−^ alone mice, suggesting that Bak deficiency does not have a significant impact in Pdx1-induced beta cell death. Additionally, our experiments show that there are no statistical differences in beta cell mass, TUNEL+ number and Ki67+ number between *Bax*^F/F^*Bak*^−/−^*Cre*^−^ and *Bax*^F/F^*Bak*^−/−^*Cre*^+^ mice (Fig. 3, *D–F*), thus indicating that even Bax deficiency in beta cells has no impact in these parameters when the mice do not have beta cell death induced by Pdx1 deficiency. Thus we would anticipate that beta cell mass, death, and proliferation would be no different between *Bax*^F/F^*Bak*^+/+^*Cre*^+^ and *Bax*^F/F^*Bak*^−/−^*Cre*^+^ mice.

In our experiments, to rule out the compensation of Bak after Bax deletion in the β-cells, *Bax*^F/F^*Bak*^−/−^*Cre*^−^ mice were chosen as control mice because Bax and Bak can compensate for each other in cells. For example, Bax and Bak can compensate for each other in MEF cells, as MEFs that express either Bax or Bak are sensitive to apoptosis induced by expression of BH3-only proteins (15–16). However, Bax Bak double knock-out MEFs are highly resistant to apoptotic cell death stimuli (17). Bak can also compensate for Bax in p53-null cells to release cytochrome *c* for the initiation of apoptosis (18). But our data indicate that Bak and Bax do not compensate for each other in beta cells.

Interestingly, Bax deficiency in *Pdx1*^+/−^*Bax*^F/F^*Bak*^−/−^*Cre*^+^ mice leads to significantly improved but not normal glucose tolerance and increased β-cell mass compared with *Bax*^F/F^*Bak*^−/−^*Cre*^−^ mice. One explanation is that although the expression of Bax in islets was reduced by 94%, the residual levels of Bax protein in islets might be enough to induce β-cell apoptosis. Another explanation is that Pdx1 deficiency could induce other forms of β-cell death other than apoptosis such as necrosis and autophagy that are not mediated by Bax expression (6, 11, 19).

In conclusion, we have shown that Bax plays a role in mediating β-cell apoptosis caused by Pdx1 deficiency. Genetic ablation of Bax rather than Bak protects β cells from apoptosis and preserves insulin secretion and β-cell mass in *Pdx1*^+/−^ mice.

## Author Contributions

K. S. P. and D. R. designed research. D. R., J. S., and L. M. performed research. D. R. and K. S. P. analyzed data and wrote the paper.

## Footnotes

3 The abbreviations used are:

Pdx1: pancreas and duodenal homeobox-1
TMRE: tetramethylrhodamine, ethyl ester
DKD: double knockdown
KD: knockdown
PI: propidium iodide.
